# Fascial Manipulation Method Is Effective in the Treatment of Myofascial Pain, but the Treatment Protocol Matters: A Randomised Control Trial—Preliminary Report

**DOI:** 10.3390/jcm11154546

**Published:** 2022-08-04

**Authors:** Mateusz Pawlukiewicz, Michał Kochan, Paweł Niewiadomy, Katarzyna Szuścik-Niewiadomy, Jakub Taradaj, Piotr Król, Michał T. Kuszewski

**Affiliations:** 1 Institute of Physioterapy and Health Sciences, The Jerzy Kukuczka Academy of Physical Education in Katowice, 40-065 Katowice, Poland; 2 Department of Balneoclimatology and Biological Regeneration, School of Health Sciences in Katowice, Medical University of Silesia in Katowice, 40-752 Katowice, Poland; 3 Department of Adapted Physical Activity and Sport, School of Health Sciences in Katowice, Medical University of Silesia in Katowice, Medyków 8, 40-752 Katowice, Poland

**Keywords:** musculoskeletal disorders, myofascial pain, fascia, fascial manipulation, centers of coordination

## Abstract

**Background:** There are many therapeutic methods targeting fascia. However, the only method whose basic assumption is to eliminate the densification of fascia is Fascial Manipulation. **Objective:** To evaluate the effectiveness of various Fascial Manipulation (FM) protocols in reducing myofascial pain. **Design:** Randomized control trial. **Subjects:** A total of 54 individuals, aged 18–29 years, with musculoskeletal pain for at least 1 week. **Methods:** The patients were divided into four groups subjected to different treatment protocols: group 1—underwent the standard FM treatment protocol (STP), group 2—modified protocol (MTP), group 3—modified protocol 2 (MTP2), and the control group (CG)—did not undergo any therapy. Each protocol involved three treatments at intervals of 7–10 days and a follow-up examination after 30 days. The outcome was pain level measured using the VAS. **Results:** In the STP, all the measurements showed a significant decrease in pain level—the mean difference was 2.077 after the first treatment, 3.462 after the third treatment and 3.385 in the follow-up. In the MTP, a significant mean difference was noted after the third treatment, 3, and in the follow up, 2.4. In the MTP2, it was noted after the third session, 2, and in the follow up, 2.25. Only the CG group did not display significant changes. **Conclusions:** FM-based therapy results in pain relief. However, there are differences in the dynamics and durability of the results depending on the chosen protocol.

## 1. Introduction

Myofascial pain (MP) is a very common problem, possibly one of the most frequent causes of muscle pain reported by patients to their GPs [1,2]. Unfortunately, many aspects related to this issue remain unclear; it is assumed, however, that the etiology of this condition involves myofascial trigger points and fascial restrictions [3,4]. As it may apply both to the torso and extremities, it is becoming an increasingly negative factor that has an impact on the daily functioning of the people affected [3,5]. The relationship between this type of pain and the central and peripheral mechanisms is widely discussed [6,7]. The central mechanism phenomenon refers to the sensitization or facilitation of the pain stimuli processing within the central nervous system, which may be caused by reorganization of neurons in a person’s brain and spinal cord [6]. In the context of this study, however, the peripheral mechanism might be more essential as it seems to be associated with fascia and its impaired functioning [7].

The Global Burden of Disease estimated that low back pain is responsible for the loss of a total of 83 million years of life, which people from around the world have had to devote to adjust to their disability (disability-adjusted life years; DALYs). In the case of neck pain and headaches (including migraines), this value is around 24 million years, and other musculoskeletal dysfunctions take 28 million years of human life around the world [8]. These statistics show how important the problem of pain is and how necessary it is to develop effective methods of managing it.

Over the recent years, there have been many publications aimed at understanding the structure and function of fascia. It turns out that fascia is a very richly innervated structure [9,10]. Due to this fact, it might play an important role in biomechanics, motor control, proprioception and postural regulation [11,12,13]. Other unique features of fascia that complement rich innervation in functional terms include: continuity—it is an uninterrupted structure over the entire human body; the ability to transmit loads—as much as about 40% of the power generated by the muscles is transmitted to the fascia via myofascial expansions; and the ability to become deformed—fascia is able to change its tension depending on an active stimulus; as a result of overload or damage, this connective tissue structure may become more tense or stiff [3,14,15]. What is more, thanks to special connections to the conjunctive tissue, the tension of one muscle can affect the muscle adjacent to it, or even the antagonists [16,17,18,19]. Several fascial layers have been distinguished, which, due to the presence of loose connective tissue between them, are able to move independently [7]. Therefore, the whole theory of engaging fascia in the peripheral mechanism responsible for myofascial pain can be considered from the mechanical as well as neurophysiological side.

Many therapeutic methods and systems target fascia. However, the only method whose basic assumption is to eliminate the densification of fascia (to restore the physiological tension of the fascial tissue and proper sliding of its layers) is Fascial Manipulation. According to this concept, it is a disorder of hyaluronic acid quality that leads to the dysfunction of the myofascial system, which, among others, results in the deterioration of joint movement and pain. The analyses made by Stecco et al. indicate that there is a strong correlation between myofascial pain and hyaluronan (HA)-rich matrix disorders [20]. An accepted pathophysiological mechanism is that inflammation in the local tissues, when paired with the movement impairment, can cause low back pain that leads to connective tissue fibrosis and decreased tissue flexibility, which exacerbates the low back pain [21,22]. However, it is assumed that, what is clinically significant, is only the densification occurring within the centers of coordination (CC), that is, contractual areas within the epimysium, where the vectors of muscle strength converge, and therefore they are in a way responsible for coordinating the activation of motor units. Thus, the author of the method suggests performing a few minutes of deep rubbing at the right sites of the human body, selected on the basis of thorough analysis and diagnosis, the aim of which is primarily to raise the temperature of the tissue. In 2013, Roman et al. proved that such an effect may actually improve the viscoelastic properties of hyaluronic acid [23], whereby the therapist’s movement must be performed in the direction in which the disorder of the fascial slide is sensed [14]. Another important aspect seems to be the reconstruction of tension balance in the myofascial system. The method distinguishes six motion sequences within three planes: sagittal (anterior movement (AN) and rear movement (RE)), frontal (lateral (LA) and medial (ME)) and transverse (internal rotation (IR) and external rotation (ER)). Thus, balancing the tension consists of choosing the centers of coordination to work on that belong to a single plane and covering at least one center of coordination from the motion sequences belonging to a given plane.

What seems to be essential is to determine whether by using this method, and theoretically improving the viscoelastic properties of fascia, we are able to alleviate pain and/or improve the functional parameters of fascia.

The aim of the study is to evaluate the effectiveness of various Fascial Manipulation (FM) protocols in reducing myofascial pain.

The research questions are:

(1) What is the effect of Fascial Manipulation therapy on the level of subjective pain sensation, as measured using the VAS?
(2) Does the protocol of FM treatment affect the patient’s response?

## 2. Materials and Methods

### 2.1. Design

The research project was designed as a randomized, double-blind trial. The Ethics Committee of the Jerzy Kukuczka Academy of Physical Education in Katowice, Poland, approved of the study (ref. 1/2017). A written informed consent was obtained from each participant prior to data collection, and the trial was registered under the number: ISRCTN13533739.

First of all, the patients were examined. The examination, carried out by diagnosticians, included history taking, pain assessment and palpatory verification based on the Fascial Manipulation method. The project coordinator then randomly assigned each patient to one of four groups (three therapeutic groups with different protocols: STP—standard treatment protocol of FM, MTP—modified FM protocol, MTP2—modified FM protocol 2; and a control group—CG—with no therapy implemented) so that the diagnosticians could select the centers of coordination in accordance with the group including particular patients. The diagnosticians indicated chosen points to the therapists, and then the points were subjected to the treatment protocol. The therapists were not familiar with the treatment methodology according to the Stecco method; therefore, they did not know to which research group a patient belonged.

**Figure 1:**
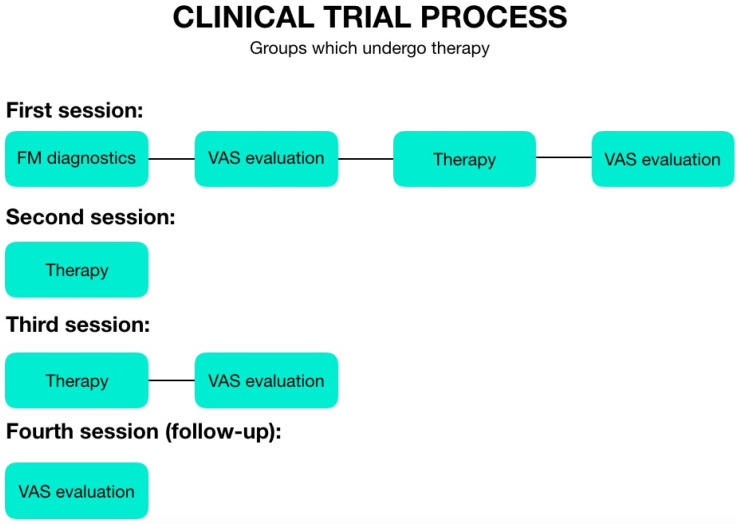
The examination scheme of the therapeutic groups.

**Figure 2:**
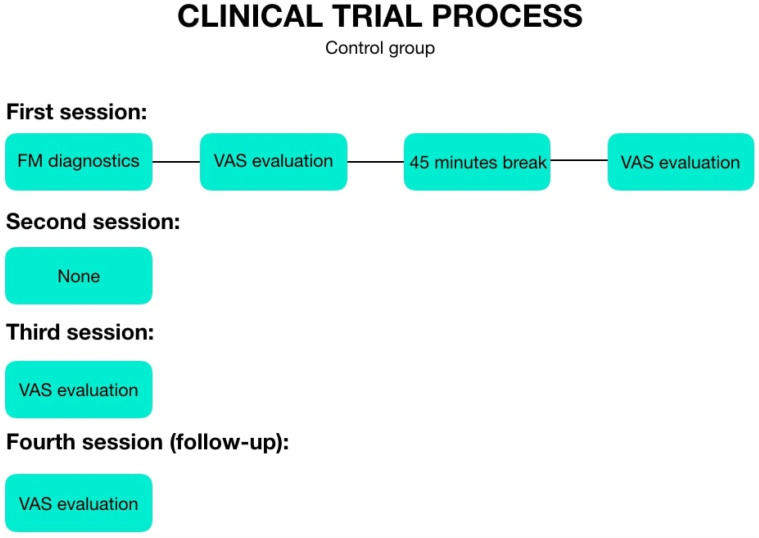
The examination scheme of the control group.

The first therapy session took place on the day of examination. After the treatment, pain was again assessed according to the VAS scale. The next meeting was arranged after 7–10 days and was only therapeutic in character. The third session took place 7–10 days after the second one and was therapeutic as well as diagnostic. Thus, the patient was first subjected to the therapy, and next the pain was assessed according to the VAS. The last meeting, the fourth one (follow-up), was arranged 30 days after the end of the therapy, where the pain assessment was carried out according to the VAS scale for the last time. As for the individuals from the control group, during the first appointment they had a 45 min break between subsequent pain tests, they did not come to the second meeting, and during the third and fourth one (follow-up), they marked their pain levels on the VAS scale. The patients were asked to sit still for 45 min. The examination schemes are presented in Figure 1 (therapeutic groups) and Figure 2 (control group).

### 2.2. Participants, Therapists and Centres

The study included 54 individuals aged 18–29 years, 31 women and 23 men. The inclusion criteria were age 18–30 years and the presence of musculoskeletal pain for at least 4 weeks. Choosing young and active adults as participants allowed the researchers to minimize the risk of chronic disorders and older age-related dysfunctions. The exclusion criteria included chronic or systemic concomitant diseases, pregnancy, the use of steroids, anti-inflammatory drugs or drugs that affect blood coagulation, the use of other therapeutic forms and serious neurological disorders. Patients with a previous history of trauma or surgery were allowed to participate in the study.

The study was conducted by a team of researchers, including a project coordinator, 2 people conducting diagnostic procedures (who have completed the second level of FM training) and 4 people responsible for performing the therapy (they were informed how to perform the therapy in particular patients, but were not familiar with the principles of Fascial Manipulation). Prior to the study, the individuals carrying out the treatment underwent a 6-month preparatory period, where they were trained in technically performing the procedure and improved their skills of working with densifications.

The participants were assigned to particular groups by means of a draw arranged before the beginning of the experiment. The allocation to one of the four groups was determined by the sequence of entering the study. In this process, the gender of the individuals was not taken into account. As a result, the groups were unequal in size and imbalanced in terms of gender distribution.

### 2.3. Intervention

The interview, data collection and palpatory verification were performed in accordance with the Fascial Manipulation method.

In all the three therapeutic groups, work with the patients involved manipulation (deep rubbing) of 6 centers of coordination, which had previously been selected by a diagnostician. Each of these sites was rubbed 3 times for 3 min (totally 9 min of stimulation of each point), in a rotational system (that is, CC1, CC2, CC3… CC6, CC1…) [24]. During each therapeutic meeting the centers of coordination stayed the same, so the therapy was repeatedly conducted on the same spots.

The first group (STP) was subjected to the therapy in accordance with the standard treatment protocol of the Fascial Manipulation method. This means that the so-called palpatory verification was followed by determining the plane (sagittal, frontal or transverse) with the largest extent of fascial disorder, that is, the plane with the most severe densifications within the centers of coordination. Then, 6 centers of coordination were selected in the most disturbed plane in such a way that the tension balance (based on points from two opposing movement sequences) was maintained. Therefore, when choosing the sagittal plane, the selected points had to include those from the anterior (AN) and rear (RE) sequences; when choosing the frontal plane, the lateral (LA) and medial (ME) sequences had to be addressed through the points covered; finally, when selecting the transverse plane, the sequence of external rotation (ER) and internal rotation (IR) had to be included.

In the second group (MTP), the therapy was carried out according to the modified FM protocol. In this group, the second most disturbed plane was chosen and CC points from two balancing sequences were also selected.

In the third group (MTP2), the modified FM 2 protocol was applied, which included centers of coordination randomly selected from all the segments subjected to the palpatory verification. In other words, the centers could belong to different zones and could not be balanced in any way.

The only side effects of the procedure were bruising and mild pain in the area of manual manipulation. These effects subsided 1–3 days after the intervention.

The last group included controls, who did not undergo any treatment.

### 2.4. Outcome Measures

The primary outcome measure was a subjectively evaluated level of pain. The tool used for this purpose was the VAS. In the course of the examination, each patient received a 10-centimetre-long line segment marked with 0 (meaning no pain), 10 (maximum level of pain) and 5 in the middle. The patients’ task was to mark the place in the segment that corresponded to the subjectively felt level of pain.

The VAS measurement was performed 4 times: before the first therapy session, directly after the first session, after the third treatment and during the follow-up.

### 2.5. Data Analysis

All the collected data were transferred to SPSS program and analyzed using the two-way repeated measures ANOVA, Box’s Test of Equality of Covariance Matrices, Multivariate Tests (Wilks’ lambda), Tests of Within-Subjects Effects (Greenhouse–Geisser), Levene’s Test of Equality of Error Variances, Tests of Between-Subjects Effects and Tukey’s test HSD. The value of *p* < 0.05 was assumed as the statistical significance level. Next, the differences between the mean VAS scores at particular assessment times within the groups were calculated. The confidence intervals were computed at the 95% level.

## 3. Results

### 3.1. Flow of Participants, Therapists and Centres through the Study

The basic anthropometric data of the examined patients from particular groups are presented in Table 1.

**Figure 3:**
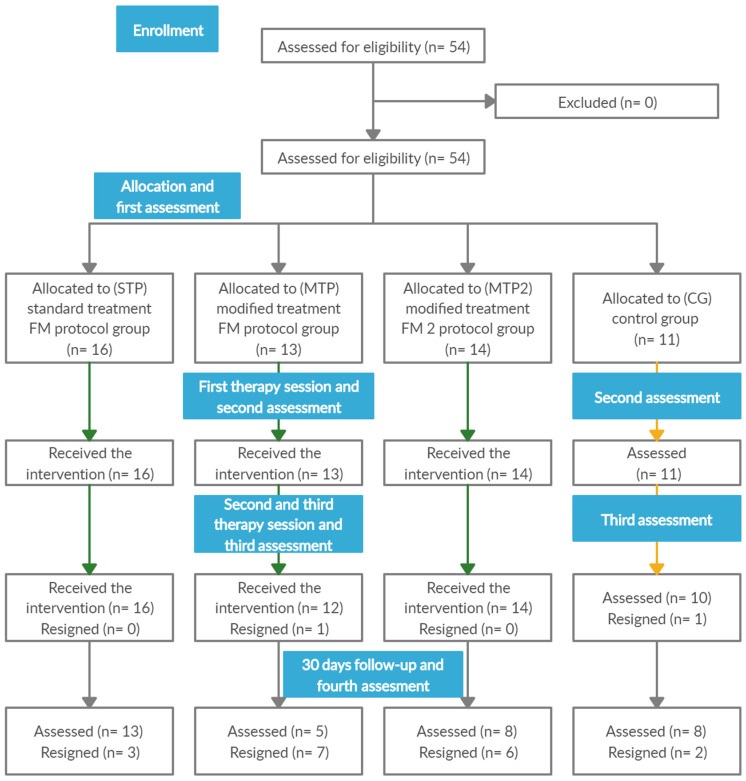
Flow of the participants through the trial.

The flow of the participants is presented in Figure 3.

### 3.2. The Assessment of the Mean Level of Pain in Particular Groups at Subsequent Measurements

**Figure 4:**
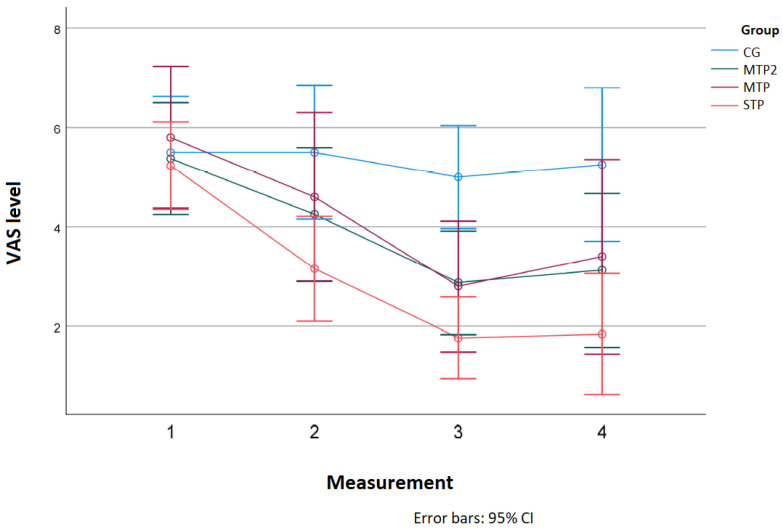
VAS levels in particular groups at subsequent measurements.

The mean VAS levels in particular groups at subsequent measurements are presented in Figure 4.

Box’s test indicated that the covariance matrices were homogenic (M-Box = 34.5; F = 1.14, *p* > 0.05). Wilks’ lambda test showed that the variation in the means on pain level (VAS) over the repeated measurement occasions itself varies as a function of treatment group membership (*p* = 0.001; the power observed for α = 0.05 was 0.933). Similar outcomes were also confirmed with the greenhouse–Geisser test.

The tests of between-subjects effects indicated that there are significant differences (*p* = 0.01; the power observed for α = 0.05 was 0.837) across the treatment groups with respect to the average VAS level (Levene’s test confirmed earlier the equality of error variances).

Comparisons of the average VAS levels (over all of the measurements) based on Tukey’s test HSD pointed out the significant difference between the STP and CG (mean difference = 2.31; SD = 0.63; *p* = 0.005); the details are presented in Table 2.

The greatest differences in the level of pain were observed in the STP group, where statistically significant changes appeared after the first therapeutic session (mean VAS difference = 2.077; *p* < 0.001), third (mean difference = 3.462; *p* < 0.001) and in the follow up (mean difference = 3.385; *p* < 0.001). In that group, the difference between the second and the third measurement was also significantly relevant (mean difference = 1.385 *; *p* = 0.012). In the MTP group, a significant difference was found between the 1–3 (VAS difference = 3; *p* < 0.001) and 1–4 measurement (VAS difference = 2.4; *p* = 0.042). In the MTP2 group, the relevant differences appeared between the measurements 1–3 (VAS difference = 2.5; *p* < 0.001) and 1–4 (VAS difference = 2.25; *p* = 0.011). The details are presented in Table 3, Figure 4.

## 4. Discussion

Several types of pain can be distinguished; this study pointed at myofascial pain, which turns out to be effectively eliminated by manual therapy techniques aimed at working with soft tissues [25,26]. These techniques include Fascial Manipulation, a method proved to be very effective in combating myofascial problems [27]. Harper et al. [27] compared the effectiveness of FM with the standard protocol treatment of low back pain, which includes mobilizations and/or manipulations of the spine joints and pelvis, mechanical traction of the lumbar spine and general treatment of soft tissues in the lumbar region. The therapy incorporating FM proved to be more efficient not only in relation to joint techniques, but also to other myofascial treatments. Despite the fact that this method turns out to be very effective in clinical conditions [28,29], to date, no one has thoroughly assessed the protocol of management in the FM method.

One of the aims of the authors’ study was to verify whether there are differences in therapeutic effects achieved by the standard protocol of FM and its modified versions. It turned out that the therapy in accordance with the protocol proposed by the author of the method ensures not only the best, but also the most dynamic improvement of the patient’s condition, which is also maintained in the follow-up examination. These observations confirm the preliminary results obtained earlier in the first examination [30]. However, the fact that statistically significant therapeutic effects were also obtained in the other two groups (particularly in the MTP2 group) seems to be most interesting.

One of the basic assumptions of using the FM technique is to treat the most disturbed planes (sagittal, frontal or transverse), and select there the most severely densified centers of coordination, in a way that enables to maintain the tension balance between the two treated sequences. It should be mentioned that, in the second group of the study (MTP), the selection of points actually occurred in accordance with the selection of the most densified CC points and the preservation of the tension balance between them, accompanied by the selection of the second most disturbed plane. Therefore, the source of improvement of the patient’s condition may be due to the improvement of tension in the myofascial system resulting from the treatment of the CC points. A situation occurred in the context of the MTP and MTP2 groups, where not only was a statistically significant improvement obtained between the examination before the therapy and after the third treatment, but the effect was also maintained in the follow-up examination. It was quite surprising for the authors; however, it may be assumed that dealing with fascia, no matter on which meridians, leads to a decrease in its tension and to reduced pain level in consequence. What is worth to emphasize is that the fastest (just after the first treatment) and the greatest effect was observed with using the original treatment protocol proposed by Stecco regarding the FM concept. It can be presumed that, due to the fact that fascia is a continuous structure, the effect will appear regardless the spot of influence, but the speed and size of positive changes could be greater, if the most disturbed areas were treated in the first place. Of course, the randomness of selection of the centers of coordination for the therapy does not exclude the possibility of targeting densified points that belong to the most disturbed plane, while maintaining the tension balance, but such a situation is unlikely to happen. It should also be emphasized that, in order to maintain the therapy repeatability and to increase the reliability of examinations, the authors decided to cover the same points in three subsequent appointments. If this project is continued in the future, this fact should be taken into consideration, as constant rotation of the centers of coordination is likely to change therapeutic effects.

Dibai-Filho et al. [31] conducted studies on patients with chronic neck pain who were assigned to one of three groups. The first group underwent manual therapy, the second group manual therapy and ultrasound in the area of the trigger point, and the third group manual therapy supplemented with diadynamic currents within the TrP. In each group, 10 therapeutic sessions were performed, 2 per week for 5 weeks. Statistical analysis showed that none of the groups achieved statistically significant improvement after the first treatment, and after 10 therapies, changes were statistically significant in all the groups, but they did not differ from each other. Compared with the results of the present study, standard manual therapy, even combined with physical stimuli, turned out to be less effective in the short run. What is more, the research required 10 therapeutic meetings, which may suggest that the therapy using FM is more profitable economically and financially.

De Meulemeester et al. [32] compared the effectiveness of using dry needling and compression therapy in the treatment of trigger points. The patients from both groups underwent four therapies, performed once a week, which focused on the treatment of TrP within particular muscles. Statistically significant changes in the severity of pain were observed only in a follow-up examination, which took place 3 months after the last treatment. However, the researchers admitted that it was not a clinically significant change. It raises the conclusion that fascial manipulations provide much faster results that are not only statistically, but also clinically, significant (pain decrease of more than 1.5 points according to the VAS) [33].

Rolfing is a method that assumes a holistic view of a human being. This assumption is very similar to that presented in FM. James et al. [34] used this method to work with patients who reported cervical spine ailments. They were subjected to 10 therapies, which is regarded as the canon of work in Rolfing. Statistically significant improvement (67%) in pain management was achieved. This efficiency is similar to that obtained in the present study (in a follow-up of the STP group), but the FM-based therapy only needed three therapeutic sessions to achieve this result.

The present study has some limitations. The main issues are unequal groups and relatively small size of samples. The reason is that the project was disturbed and interrupted by the COVID-19 pandemic. Unfortunately, after the pandemic, some people involved in the study (therapists and a diagnostician) could not continue the research. However, the results obtained to date were so promising that we considered that it would be valuable to present them as a preliminary report.

## 5. Conclusions

The Fascial Manipulation method leads to myofascial pain reduction. Compared to the therapeutic effects achieved in the modified treatment protocol groups and in the control group, conducting the therapy in accordance with the standard treatment protocol of the FM method is the most effective way. Nevertheless, in view of obtaining statistically significant changes in the modified treatment protocols groups, further research is required to determine which mechanisms lie behind the therapeutic effects in these groups. In addition, compared to the studies cited in the discussion, therapy using the Fascial Manipulation method may guarantee effects that are faster, more significant for the patient and less costly.

## Figures and Tables

**Table 1:** Basic anthropometric data of the participants from particular groups.

Group	Sex		Age			Height [cm]			Weight [kg]		
	F (%)	M (%)	x	Min–Max	SD	x	Min–Max	SD	x	Min–Max	SD
STP	10 (63)	6 (37)	22	18–29	2.8	170	160–178	8.7	65	53–70	8.2
MTP	5 (38)	8 (62)	23	20–28	2.4	176	160–186	11.1	71	54–85	16
MTP2	8 (57)	6 (43)	23	22–29	2.3	170	160–185	8.75	65	55–85	12
CG	8 (73)	3 (27)	22	20–25	1.4	174	163–190	10.3	69	54–80	10

**Table 2:** Mean VAS differences between groups over all of the measurements based on Tukey’s test HSD.

Group (A)	Referred to Group (B)	Mean Difference (A–B)	SD	*p*-Value	95% Confidence Interval	
					Lower Bound	Upper Bound
STP	MTP	−1.15	0.739	0.418	−3.16	0.86
	MTP2	−0.91	0.631	0.488	−2.62	0.81
	CG	−2.31 *	0.631	0.005	−4.03	−0.60
MTP	STP	1.15	0.739	0.418	−0.86	3.16
	MTP2	0.24	0.801	0.990	−1.93	2.42
	CG	−1.16	0.801	0.478	−3.34	1.01
MTP2	STP	0.91	0.631	0.488	−0.81	2.62
	MTP	−0.24	0.801	0.990	−2.42	1.93
	CG	−1.41	0.702	0.210	−3.32	0.50
CG	STP	2.31 *	0.631	0.005	0.60	4.03
	MTP	1.16	0.801	0.478	−1.01	3.34
	MTP2	1.41	0.702	0.210	−0.50	3.32

* The mean difference is significant at the 0.05 level.

**Table 3:** Mean VAS differences between measurements in particular groups based on Tukey’s test HSD.

Group	Measurement (A)	Referred to Measurement (B)	Mean Difference (A–B)	SD	*p*-Value	95% Confidence Interval	
						Lower Bound	Upper Bound
STP	1	2	2.077 *	0.330	<0.001	1.143	3.010
		3	3.462 *	0.369	<0.001	2.418	4.505
		4	3.385 *	0.515	<0.001	1.931	4.838
	2	3	1.385 *	0.411	0.012	0.225	2.544
		4	1.308	0.664	0.349	−0.568	3.183
	3	4	−0.077	0.562	1.000	−1.664	1.510
MTP	1	2	1.200	0.533	0.191	−0.305	2.705
		3	3.000 *	0.596	<0.001	1.317	4.683
		4	2.400 *	0.830	0.042	0.056	4.744
	2	3	1.800	0.662	0.065	−0.070	3.670
		4	1.200	1.071	1.000	−1.824	4.224
	3	4	−0.600	0.906	1.000	−3.159	1.959
MTP2	1	2	1.125	0.421	0.073	−0.065	2.315
		3	2.500 *	0.471	<0.001	1.170	3.830
		4	2.250 *	0.656	0.011	0.397	4.103
	2	3	1.375	0.523	0.081	−0.103	2.853
		4	1.125	0.846	1.000	−1.266	3.516
	3	4	−0.250	0.716	1.000	−2.273	1.773
CG	1	2	−8.882	0.421	1.000	−1.190	1.190
		3	0.500	0.471	1.000	−0.830	1.830
		4	0.250	0.656	1.000	−1.603	2.103
	2	3	0.500	0.523	1.000	−0.978	1.978
		4	0.250	0.846	1.000	−2.141	2.641
	3	4	−0.250	0.716	1.000	−2.273	1.773

* The mean difference is significant at the 0.05 level.

## Data Availability

The data will be made available in accordance with the policy of The Jerzy Kukuczka Academy of Physical Education.
